# Efficacy and Safety of Tranexamic Acid in Pediatric Patients Undergoing Cardiac Surgery: A Single-Center Experience

**DOI:** 10.3389/fped.2019.00181

**Published:** 2019-05-07

**Authors:** Yu Zhang, Xue Zhang, Yang Wang, Jia Shi, Su Yuan, Fujian Duan, Yuefu Wang, Zhe Zhang, Yuan Jia, Junsong Gong, Lihuan Li, Fuxia Yan

**Affiliations:** ^1^Department of Anesthesiology, National Center for Cardiovascular Diseases and Fuwai Hospital, Chinese Academy of Medical Sciences and Peking Union Medical College, Beijing, China; ^2^Department of Information Center, National Center for Cardiovascular Diseases and Fuwai Hospital, Chinese Academy of Medical Sciences and Peking Union Medical College, Beijing, China; ^3^Department of Medical Research & Biometrics Center, National Center for Cardiovascular Diseases and Fuwai Hospital, Chinese Academy of Medical Sciences and Peking Union Medical College, Beijing, China

**Keywords:** congenital heart disease, tranexamic acid, infant, cyanosis, safety, blood loss

## Abstract

**Aims:** This study evaluated the efficacy and safety of tranexamic acid (TXA) undergoing cardiac surgery.

**Methods:** Using a retrospective cohort study design, 2,026 consecutive pediatric patients who underwent surgical repair of atrial or ventricular septal defect or complete repair of Tetralogy of Fallot were included, and divided into a control group and a TXA group.

**Results:** Compared with that in the control group, there were statistically significant reduction of both the 12-h and total postoperative blood loss in the TXA group [6.573 ± 0.144 vs. 5.499 ± 0.133 ml kg^−1^, mean difference (MD) 1.074 ml kg^−1^, *p* < 0.001; 12.183 ± 0.298 vs. 9.973 ± 0.276 ml kg^−1^, MD, 2.210 ml kg^−1^, *p* < 0.001]. There was a statistically significant reduction of the MD of 12-h postoperative blood loss due to TXA in patients aged < 1 year compared with that in patients aged ≥1 year (MD, 1.544 vs. 0.681 ml kg-1, *P* = 0.007). There were statistically significant reduction of the MD of both the 12-h and total postoperative blood loss due to TXA in patients weighing < 10 kg compared with that in patients weighing ≥10 kg (MD, 1.542 vs. 0.456 ml kg-1, *P* < 0.001, and MD, 2.195 vs. 0.929 ml kg-1, *P* = 0.036, respectively). There was a statistically significant reduction of the MD of total postoperative blood loss due to TXA in cyanotic patients compared with that in acyanotic patients (MD, 3.381 vs. 1.038 ml kg^−1^, *P* = 0.002). There was no significant difference in the postoperative volume or exposure of allogeneic transfusion, in-hospital morbidity or mortality between the groups.

**Conclusions:** TXA took effects in reduction of postoperative blood loss but not the allogeneic transfusion requirement in pediatric patients undergoing cardiac surgery, particularly in infants weighing < 10 kg and cyanotic children. Moreover, the study suggested the use of TXA was safe in pediatric cardiac surgery.

## Introduction

Congenital heart disease (CHD) has been associated with abnormal coagulation, including low levels of fibrinogen and platelet dysfunction. Moreover, during the process of cardiopulmonary bypass (CPB), exposure of blood to artificial surfaces and hemodilution of blood due to priming volume caused activation of platelets, coagulation, and fibrinolysis, leading to a decrease in platelet number and function, and a reduction in fibrinogen levels (1). Therefore, pediatric patients undergoing cardiac surgery are at high risk of excessive bleeding and needing blood transfusion, which may increase postoperative morbidity and mortality (2). Since the suspension of aprotinin in 2008, tranexamic acid (TXA) has become the main antifibrinolytic agent to prevent blood loss in cardiac surgery (3). However, as the guideline points out, clinical studies on the use of TXA in pediatric cardiac surgery have been limited by small sample sizes and marked heterogeneity in the data (4). Moreover, the effect of TXA in infants (age 31 days−1 year) weighing < 10 kg and pediatric patients with cyanosis, who are at increased risk of bleeding due to the specific hemostatic characteristics, remained uncertain.

As a result of aprotinin story, TXA should be given more attention considering the adverse events. Pasquali et al suggested that TXA was associated with significantly reduced mortality compared with aprotinin in pediatric cardiac surgery (5). Unfortunately, however, recent clinical trials and meta-analyses have shown a dose-dependent association between TXA and the risk of seizures in adults who undergo cardiac surgery (6–8). Retrospective studies revealed that TXA use was associated with a significantly increased risk of seizures in pediatric cardiac surgery (9, 10). TXA associated seizures may worsen the prognosis in pediatric cardiac surgery (11). Therefore, safety evaluations of TXA remain sparse in pediatric patients undergoing cardiac surgery.

The aim of this study was to evaluate the efficacy and safety of TXA in pediatric patients undergoing cardiac surgery.

## Materials and Methods

### Patients and Study Design

This study was a retrospective, single-center, cohort study. The study protocol was approved by the institutional review board of Fuwai Hospital. The requirement for written informed consent was waived by the board. Two thousand and twenty six consecutive pediatric patients aged 31 to 12 years who underwent primary surgical repair of acyanotic CHD, i.e., atrial or ventricular septal defect, or complete repair for cyanotic CHD, i.e., Tetralogy of Fallot, at Fuwai Hospital in Beijing, China, between January 1, 2009 and December 31, 2010, were eligible for inclusion. The patients were divided into a control group that did not receive an antifibrinolytic agent during surgery (*n* = 1056) and a TXA group that was intravenously administered TXA after induction with a pump by maintenance at 15 mg/kg/h until termination of CPB (*n* = 970).

### Perioperative Management

The standard surgical and anesthetic management techniques used in patients with atrial or ventricular septal defect and Tetralogy of Fallot were followed. Systemic anticoagulation was achieved using heparin 400 U/kg, with additional doses administered to maintain an activated clotting time >480 s. Priming volumes in CPB circuit depended on pediatric patients' body weights. The CPB circuit was primed with crystalloid and colloid. Packed red blood cells were also added to the prime to achieve a hematocrit level of >25% if the body weight was < 10 kg. CPB was used and modified ultrafiltration was performed after separation from CPB. Protamine was administered at a protamine to heparin ratio of 1–1.2 to 1. No additional hemostatic agents were administered intraoperatively.

### Measurements

The primary outcome was postoperative blood loss. The nurse in the ICU recorded postoperative volume of pericardial and mediastinal fluid collected via a drainage tube per hour. Twelve-hour postoperative blood loss was measured as the accumulated volume of pericardial and mediastinal fluid collected via a drainage tube during the first 12 h after surgery and total postoperative blood loss was that measured before removal of the tubes. The secondary efficacy outcomes were the intraoperative blood loss, the volume and exposure of allogeneic transfusion after termination of CPB. The threshold for red blood cell transfusion was a hemoglobin concentration of < 100 g l^−1^ after termination of CPB. The indication for fresh frozen plasma was a requirement for clotting factors based on the results of coagulation tests. Concentrated platelets were administered at the discretion of the attending surgeon. The secondary safety outcomes were postoperative morbidity and mortality. Morbidity parameters included stroke, seizure, renal failure, deep venous thrombosis, use of extracorporeal membrane oxygenation, reoperation for bleeding, and prolonged mechanical ventilation. Stroke was diagnosed as a new focal neurologic deficit lasting >24 h or leading to earlier death and was confirmed by computed tomography or magnetic resonance imaging showing cerebral infarction or hemorrhage (12). Seizure was identified as a new-onset neuropsychiatric disorder with increased motor activity or an agitated or hyperactive state (12). Renal failure was defined as a need for postoperative peritoneal dialysis (9). Deep venous thrombosis was confirmed by clinical symptoms and venous Doppler ultrasonography findings (9). Reoperation for bleeding was performed when massive bleeding occurred with a drainage rate >10% of total blood volume per hour for up to 2 h or if cardiac tamponade was detected. Prolonged mechanical ventilation was defined as postoperative mechanical ventilation lasting longer than 72 h (13). Additionally, other study variables were the maximum creatinine value in the first 48 h postoperatively, length of stay in the intensive care unit, and duration of hospital stay.

### Statistical Analysis

Normally distributed continuous variables are shown as the mean and standard deviation and were compared using the Student's *t*-test. Non-normally distributed continuous variables are shown as the median and interquartile range and were compared using the Wilcoxon-Mann-Whitney test. Categorical variables are presented as the frequency and percentage and were compared using the chi-square or Fisher's exact test. A general linear regression model was used to analyse the maximum hemoglobin level in the first 48 h postoperatively, blood loss, allogeneic transfusion volume. The mean difference (MD) and 95% confidence interval (CI) were calculated. A multivariate logistic regression model was used to analyse allogeneic transfusion exposure. The odds ratio and 95% CI were calculated. Covariates were included into the regression analysis if the baseline confounders showed significant difference between the two groups or exactly affected the pharmacokinetics of TXA (including age, weight, type of surgery, CPB time, aortic cross-clamp time and surgical time) (14). All tests were two-sided, and a probability value < 0.05 was considered statistically significant. All statistical analyses were performed using SAS version 9.2 (SAS Institute Inc., Cary, NC, USA).

## Results

### Perioperative Data

There were 1,056 patients in the control group and 970 in the TXA group. The patient characteristics, including patient gender, age, height, and weight, were comparable between the two groups. There were 584 infants aged < 1 year and 884 pediatric patients weighing < 10 kg. The preoperative data, including for hemoglobin, platelets, prothrombin time, prothrombin time activity, international normalized ratio, and creatinine, were comparable between the groups. An atrial septal defect was repaired in 161 patients (15.24%) in the control group and 118 (12.16%) in the TXA group and a ventricular septal defect in 735 (69.60%) and 626 (64.54%), respectively. One hundred and sixty one patients (15.24%) in the control group and 225 (23.20%) in the TXA group underwent complete repair for tetralogy of Fallot. Compared with the control group, the TXA group had a significantly longer CPB time (66.62 ± 32.137 vs. 75.64 ± 37.104 min, *P* < 0.001), aortic cross-clamp time (41.56 ± 24.731 vs. 48.31 ± 27.840 min, *P* < 0.001), and surgical time (147.87 ± 42.394 vs. 159.59 ± 48.668 min, *P* < 0.001). The perioperative data for cyanotic patients were comparable between the two groups (Table 1).

**Table 1:** Perioperative data.

	**The control group (*n* = 1,056)**	**The TXA group (*n* = 970)**	***P*-value**
**Patient characteristics**			
Male gender(%)	588 (55.68)	581 (59.90)	0.059
Age (years)	2.553 ± 1.938	2.483 ± 2.039	0.423
Age in TOF (years)	1.626 ± 1.411	1.417 ± 1.135	0.122
Infants (< 1 year, %)	289 (27.37)	295 (30.41)	0.131
Height (cm)	87.51 ± 17.138	86.33 ± 17.776	0.129
Weight (kg)	12.37 ± 4.834	12.14 ± 5.036	0.283
Weight in TOF (kg)	9.83 ± 3.103	9.67 ± 2.599	0.597
Weight (< 10 kg, %)	430 (40.72)	454 (46.80)	0.006
**Preoperative data**			
Hemoglobin (g l^−1^)	120.22 ± 15.876	121.31 ± 17.540	0.286
Hemoglobin in TOF (g l^−1^)	133.53 ± 24.662	132.82 ± 25.168	0.840
Platelets (10^9^ l^−1^)	304.77 ± 81.762	299.79 ± 90.578	0.373
PT (s)	14.08 ± 1.253	13.93 ± 1.133	0.171
PTA (%)	85.46 ± 12.099	85.16 ± 8.921	0.769
INR	1.09 ± 0.139	1.08 ± 0.118	0.505
Creatinine (umol l^−1^)	30.03 ± 12.000	31.63 ± 12.524	0.107
**Surgical data**			
Type of surgery (%)			< 0.001
ASD	161 (15.24)	118 (12.16)	
VSD	735 (69.60)	626 (64.54)	
TOF	161 (15.25)	225 (23.20)	
CPB time (min)	66.62 ± 32.137	75.64 ± 37.104	< 0.001
CPB time in TOF (min)	112.04 ± 37.364	115.60 ± 40.281	0.379
Aortic cross-clamp time (min)	41.56 ± 24.731	48.31 ± 27.840	< 0.001
Aortic cross-clamp time in TOF (min)	76.35 ± 27.919	78.49 ± 28.587	0.466
Surgical time (min)	147.87 ± 42.394	159.59 ± 48.668	< 0.001
Surgical time in TOF (min)	200.19 ± 48.463	203.29 ± 55.354	0.569

*Data are presented as incidence (%) or mean ± SD. TXA, tranexamic acid; TOF, tetralogy of Fallot; PT, prothrombin time; PTA, prothrombin time activity; INR, international normalized ratio; ASD, atrial septal defect; VSD, ventricular septal defect; CPB, cardiopulmonary bypass*.

### Blood Loss and Transfusion Outcomes

There was no significant difference in intraoperative blood loss between the two groups. Compared with that in the control group, there was a statistically significant reduction of the 12-h postoperative blood loss in the TXA group (6.573 ± 0.144 vs. 5.499 ± 0.133 ml kg^−1^; MD, 1.074 ml kg^−1^; 95% CI, 0.710 to 1.438 ml kg^−1^; *p* < 0.001). Compared with that in the control group, there was a statistically significant reduction of the total postoperative blood loss in the TXA group (12.183 ± 0.298 vs. 9.973 ± 0.276 ml kg^−1^; MD, 2.210 ml kg^−1^; 95% CI, 1.456 to 2.964 ml kg^−1^; *p* < 0.001). There was no significant difference in the maximum hemoglobin level in the first 48 h postoperatively or in the allogeneic transfusion volume or exposure after termination of CPB (Table 2).

**Table 2:** Blood loss and transfusion outcomes.

	**The control group (*n* = 1,056)**	**The TXA group (*n* = 970)**	***P*-value**	**Mean difference (95%CI)**	**Odds ratio (95%CI)**
**BLOOD LOSS**					
Intraoperative blood loss (ml kg^−1^)	5.316 ± 0.090	5.224 ± 0.083	0.428	0.092 (−0.135 to 0.319)	–
12-h postoperative blood loss (ml kg^−1^)	6.573 ± 0.144	5.499 ± 0.133	< 0.001	1.074 (0.710–1.438)	–
Total postoperative blood loss (ml kg^−1^)	12.183 ± 0.298	9.973 ± 0.276	< 0.001	2.210 (1.456–2.964)	–
Maximum hemoglobin (g l^−1^ 48 h^−1^)	127.042 ± 0.837	128.398 ± 0.652	0.180	−1.355 (−3.338 to 0.627)	–
**ALLOGENEIC TRANSFUSION**					
RBC (u)	0.185 ± 0.016	0.177 ± 0.016	0.715	0.008 (−0.036 to 0.053)	–
FFP (ml)	34.601 ± 1.897	32.578 ± 1.980	0.463	2.022 (−3.382 to 7.427)	–
Platelets (u)	0.08 ± 0.03	0.12 ± 0.03	0.329	−0.04 (−0.13 to 0.05)	–
**PATIENTS EXPOSED (%)**					
RBC	161 (15.25)	148 (15.26)	0.084	–	0.794 (0.611–1.031)
FFP	234 (22.16)	253 (26.08)	0.134	–	0.818 (0.629–1.064)
Platelets	6 (0.57)	14 (1.44)	0.364	–	1.592 (0.584–4.341)

*Data are presented as mean ± SD or incidence (%). TXA, tranexamic acid; RBC, red blood cells; FFP, fresh frozen plasma*.

### Subgroup Analysis

The 12-h postoperative blood loss in patients aged < 1 year was 7.309 ± 0.213 ml kg^−1^ in the control group and 5.765 ± 0.216 ml kg^−1^ in the TXA group and that for patients aged ≥1 year was 5.647 ± 0.126 ml kg^−1^ and 4.966 ± 0.130 ml kg^−1^, respectively. There was a statistically significant reduction of the MD of 12-h postoperative blood loss due to TXA in patients aged < 1 year compared with that in patients aged ≥1 year (MD, 1.544 vs. 0.681 ml kg^−1^, *P* = 0.007). For total postoperative blood loss, there was no significant difference of the MD of total postoperative blood loss due to TXA between age and treatment with TXA (MD 1.764 vs. 1.367 ml kg^−1^, *P* = 0.547).

The 12-h postoperative blood loss in patients weighing < 10 kg was 7.692 ± 0.171 ml kg^−1^ in the control group and 6.150 ± 0.171 ml kg^−1^ in the TXA group, and that for patients weighing ≥10 kg was 4.932 ± 0.142 ml kg^−1^ and 4.476 ± 0.154 ml kg^−1^, respectively. There was a statistically significant reduction of the MD of 12-h postoperative blood loss due to TXA in patients weighing < 10 kg compared with that in patients weighing ≥10 kg (MD, 1.542 vs. 0.456 ml kg^−1^, *P* < 0.001). The total postoperative blood loss in patients weighing < 10 kg was 13.173 ± 0.356 ml kg^−1^ in the control group and 10.978 ± 0.356 ml kg^−1^ in the TXA group, and that for patients weighing ≥10 kg was 9.354 ± 0.295 ml kg^−1^ and 8.425 ± 0.320 ml kg^−1^, respectively. There was a statistically significant reduction of the MD of total postoperative blood loss due to TXA in patients weighing < 10 kg compared with that in patients weighing ≥10 kg (MD, 2.195 vs. 0.929 ml kg^−1^, *P* = 0.036).

There was no significant difference of the MD of 12-h postoperative blood loss due to TXA between cyanotic status and TXA treatment (MD 1.304 vs. 0.844 ml kg^−1^, *P* = 0.214). The total postoperative blood loss in cyanotic patients was 14.013 ± 0.577 ml kg^−1^ in the control group and 10.632 ± 0.514 ml kg^−1^ in the TXA group, and that for acyanotic patients was 10.352 ± 0.235 and 9.314 ± 0.251 ml kg^−1^, respectively. There was a statistically significant reduction of the MD of total postoperative blood loss due to TXA in cyanotic patients compared with that in acyanotic patients (MD, 3.381 vs. 1.038 ml kg^−1^, *P* = 0.002) (Table 3).

**Table 3:** Interactions of postoperative blood loss with age, weight, cyanotic status, and administration of tranexamic acid.

	**The control group (*n* = 1,056)**	**The TXA group (*n* = 970)**	**Mean difference**	***P*-value**
**12-H Postoperative Blood Loss (Ml Kg**^**−1**^**)**				
Age (< 1 year)	7.309 ± 0.213	5.765 ± 0.216	1.544	0.007
Age (≥1 year)	5.647 ± 0.126	4.966 ± 0.130	0.681	
Weight (< 10 kg)	7.692 ± 0.171	6.150 ± 0.171	1.542	< 0.001
Weight (≥10 kg)	4.932 ± 0.142	4.476 ± 0.154	0.456	
Cyanosis	7.289 ± 0.279	5.985 ± 0.248	1.304	0.214
Acyanosis	5.858 ± 0.114	5.014 ± 0.121	0.844	
**TOTAL POSTOPERATIVE BLOOD LOSS (ML KG**^**−1**^**)**				
Age (< 1 year)	12.866 ± 0.443	11.102 ± 0.449	1.764	0.547
Age (≥1 year)	10.249 ± 0.261	8.882 ± 0.271	1.367	
Weight (< 10 kg)	13.173 ± 0.356	10.978 ± 0.356	2.195	0.036
Weight (≥10 kg)	9.354 ± 0.295	8.425 ± 0.320	0.929	
Cyanosis	14.013 ± 0.577	10.632 ± 0.514	3.381	0.002
Acyanosis	10.352 ± 0.235	9.314 ± 0.251	1.038	

*Data are presented as mean ± SD. TXA, tranexamic acid*.

### Postoperative Outcomes

There was no in-hospital mortality in the control group. One patient (0.10%) in the TXA group died of multiple organ failure (*P* = 0.309). There was no significant difference in in-hospital morbidity (including seizure, stroke, renal failure, deep venous thrombosis, use of extracorporeal membrane oxygenation, reoperation for bleeding, and prolonged mechanical ventilation) between the groups. There was also no significant difference in the maximum creatinine value in the first 48 h postoperatively between the two groups. The postoperative stays in the intensive care unit and in hospital were comparable between the two groups (Table 4).

**Table 4:** Postoperative outcomes.

	**The control group (*n* = 1,056)**	**The TXA group (*n* = 970)**	***P*-value**
Mortality at discharge (%)	0	1 (0.10)	0.297
**MORBIDITY AT DISCHARGE (%)**			
Seizure	0	0	>0.99
Stroke	1 (0.09)	0	0.338
Maximum creatinine (umol l^−1^ 48 h^−1^)	39.85 ± 12.939	40.97 ± 13.383	0.307
Renal failure	2 (0.19)	1 (0.10)	0.614
Deep venous thrombosis	1 (0.09)	0	0.338
ECMO	0	1 (0.10)	0.297
Reoperation for bleeding	3 (0.28)	2 (0.21)	0.724
Prolonged mechanical ventilation	16 (1.52)	15 (1.55)	0.954
**POSTOPERATIVE TIME COURSE**			
ICU stay (days)	1 (1–2)	1(1–2)	0.539
Hospital length of stay (days)	7.403 ± 3.698	7.640 ± 4.122	0.172

*Data are presented as incidence (%), mean ± SD or median (IQR). TXA, tranexamic acid; ECMO, extracorporeal membrane oxygenation; ICU, intensive care unit*.

## Discussion

The results of our study suggested that TXA took effects in reduction of postoperative blood loss but not the allogeneic transfusion requirement in pediatric patients undergoing cardiac surgery, particularly in infants weighing < 10 kg and children with cyanosis. Moreover, the study suggested that the use of TXA was safe in pediatric cardiac surgery.

As we know, aprotinin was just withdrawn from the market in 2008, and the clinical blood protection was still in a transitional period during 2009-2010. The study on the effects of TXA in pediatric cardiac surgery was limited at that time. Therefore, the anesthesiologist decided whether to apply TXA for blood protection in pediatric cardiac surgery according to their personal experience. The patients in our study were intravenously administered TXA after induction with a pump by maintenance at 15 mg/kg/h until termination of CPB as the method in US Children's Hospitals. At present, there is wide variation in the dosage of TXA recommended for use during pediatric cardiac surgery (Loading doses of TXA ranged from 10 to 100 mg/kg and maintenance doses ranged from 1 to 15 mg/kg.h.) (15). Most studies reported late postoperative drain losses to evaluate postoperative blood loss; however, some at this time post-surgery these might be a mixture of blood and serosanguinous due to the post-bypass inflammatory response (1). Therefore, we not only recorded late postoperative drain losses, but also recorded early postoperative drain losses to exclude the influence of serosanguinous. TXA took effects in reduction of the 12-h postoperative blood loss by 1.074 ml kg^−1^ and the total postoperative blood loss by 2.210 ml kg^−1^, but had no effect on the requirement for allogeneic transfusion. These results are comparable with those of some meta-analyses published in recent years (16, 17). However, the meta-analysis published by Faraoni et al. (17) showed that TXA reduced both postoperative blood loss and the requirement for allogeneic transfusion in pediatric cardiac surgery. The inconsistencies in the findings between our study and their meta-analysis likely reflect the type of race selected and the dosage regimen of TXA used. Our results are consistent with those of a randomized trial showing that TXA took effects in reduction of blood loss in pediatric cardiac surgery but not the transfusion requirement (18).

In the present study, TXA took effects in reduction of both 12-h and total postoperative blood loss in pediatric patients undergoing cardiac surgery, especially in infants weighing < 10 kg and in cyanotic children. Infants weighing < 10 kg are at particularly high risk of postoperative blood loss because of their immature coagulation systems, lower levels of fibrinogen, and the mismatch between the CPB priming volume and the infants' blood volume, resulting in hemodilution of up to 50-100%, which activates the inflammatory cascade and increases fibrinolytic activity (1). Moreover, cyanotic children undergoing cardiac surgery reportedly have significant preoperative coagulation anomalies and require more fibrinogen supplementation postoperatively (19). Therefore, we considered that it might be related to the antifibrinolytic, anti-platelet activation, and anti-inflammatory effects of TXA (20, 21), which might be more beneficial in infants weighing < 10 kg and pediatric patients with cyanosis undergoing cardiac surgery, who are at high risk of postoperative blood loss due to the specific hemostatic characteristics. As we all know, massive hemorrhage and allogeneic blood transfusion increased postoperative morbidity and mortality. If perioperative blood transfusion cannot be reduced by TXA, but only the postoperative thoracic mediastinal drainage fluid can be reduced, giving TXA in pediatric cardiac surgery at potential risk is worth considering. Based on the above results, it is not recommended to use TXA in children with simple CHD. TXA is more suitable for infants weighing < 10 kg and pediatric patients with cyanosis undergoing cardiac surgery. Therefore, the specific antifibrinolytic regimens to these patients undergoing cardiac surgery require further study.

Actually, Maeda et al. (10) reported TXA use was associated with a significantly increased risk of seizures. However, accurate data on doses of TXA were not available in the database. They found 0.2% seizure in non-TXA group and 1.6% seizure in TXA group. According to their results, more than 710 patients per group are needed to evaluate side effects related to TXA administration. This retrospective, single-center, cohort study assessed the benefit of TXA in a very large number of consecutive pediatric patients (*n* = 2026) undergoing cardiac surgery, which was powerful to explain the safety of TXA. In our present study, no seizures occurred in either study group after pediatric cardiac surgery. This might be the dosage regimen in our center, which was intravenously administered TXA after induction with a pump by maintenance at 15 mg/kg/h until termination of CPB, without the central nervous system damaged due to excessive TXA concentration in the brain from a bolus injection or a high dose of TXA. Our present data showed that there was no correlation noted between TXA and postoperative morbidity and mortality, which is consistent with previous prospective and retrospective reports (10, 17, 22).

This study has several limitations. First, the present study is a retrospective single-center design. Therefore, the potential problems of a non-randomized study may remain despite multivariate adjustment being used to reduce overt bias. Second, the present study excluded some high-risk patients, such as complex CHD. Therefore, the sample population was not representative of all patients in our institution. The safety and efficacy of TXA during pediatric cardiac surgery for these high-risk patients remains unexplored. Third, although the data presented in this study could be dated back to 2009 and 2010, the CHD in this study was relatively simple and the methods of surgery, anesthesia and CPB have not changed much. In addition, aprotinin was just withdrawn from the market at this stage, and there was no other hemostatic drugs except for TXA at that time, which reduced the heterogeneity of the study.

## Conclusions

In this analysis of 2,026 consecutive pediatric patients undergoing primary cardiac surgery, TXA took effects in reduction of postoperative blood loss but not the allogeneic transfusion requirement, particularly in infants weighing < 10 kg and children with cyanosis. Moreover, the study suggested that the use of TXA was safe in pediatric cardiac surgery. However, large, multicenter, prospective randomized controlled trials are needed to evaluate the benefits of TXA and the most appropriate way of administering it in infants weighing < 10 kg and children with cyanosis undergoing cardiac surgery.

## Ethics Statement

The study protocol was approved by the institutional review board of Fuwai Hospital. The requirement for written informed consent was waived by the board.

## Author Contributions

YZ responsible for interpretation of the data, statistical analysis, drafting of the manuscript, and approval of the final version to be published. XZ, YaW, JS, SY, FD, YuW, ZZ, YJ, JG, LL, and FY were responsible for study conception, data collection and interpretation, revision of the manuscript, and approval of the final version to be published. All authors read and approved the final manuscript.

### Conflict of Interest Statement

The authors declare that the research was conducted in the absence of any commercial or financial relationships that could be construed as a potential conflict of interest.
